# The Mosquito-Malarial Fever and Quinine

**Published:** 1901-02

**Authors:** 


### THE MOSQUITO-MALARIAL FEVER AND QUININE.

We have from time to time kept our readers informed of the progress of what was at one time a most interesting hypothesis in regard to the dissemination of malarial fever,—namely, that it was caused by the inoculation of human beings through the agency of mosquitoes which had previously been infected by the malarial germ; and it will be remembered that we published in the original and editorial columns of the *Gazette,* towards the close of 1899, an interesting paper from the Hygienic Institute in Rome. detailing experiments which had been made to discover the best means of destroying the mosquito in its early development so that large tracts of land at one time almost uninhabitable because of these pests might become entirely free from this objection. Largely through the splendid enthusiasm of Dr. Patrick Manson, so well known to many of us because of his investigations into tropical diseases, and also through the interest of Grassi, Bignami, and Bastianelli, certain experiments have been carried out during the past year, and more particularly within the last few months, which are epoch-making in their results.

The most recent and one of the most important contributions to this subject with which we are acquainted is the paper which appears in the *British Medical Journal* and in *The Lancet* of September 29, 1900, by Dr. Manson, in which he gives in detail the interesting measures which were taken for obtaining mosquitoes that had had an opportunity to become infected in such a manner that they were capable of transferring the infection to a human being. In order that his experiment might be startling in its effect, with the object of making the laity take an interest in this matter as well as the profession, he arranged that mosquitoes should be captured in Italy; that they should feed upon the blood of a malarial patient; that they should then be carried to the centre of London, where there is no malaria, and that a volunteer should expose himself to their bites; feeling confident that this volunteer would thereby become inoculated, being, of course, careful that the volunteer was a person who by no possible chance could ever have been subjected to malarial infection previously.

Bignami and Bastianelli sent Manson relays of infected mosquitoes from Rome in cages well devised for their safe transportation, care being taken that these mosquitoes were fed upon patients suffering from pure benign tertian infection, since it was recognized that to use mosquitoes which had been infected by the æstivo-autumnal parasite might produce serious pathological changes before the patient could be successfully relieved. The mosquitoes were forwarded to the London School of Tropical Medicine through the British Embassy at Rome. Some of them died on the way, and some died after their arrival. But a fair proportion survived and appeared to be healthy and vigorous. With true Spartan enthusiasm, Dr. Manson now arranged that his son, P. Thurburn Manson, in Guy's Hospital, a man twenty-three years of age, should expose his hand to the infected mosquitoes. As a result Mr. Manson suffered from the characteristic symptoms of malarial fever, all of which symptoms disappeared when quinine was properly administered, and in his blood prior to the administration of the quinine Patrick Manson and other competent observers were able to discover the presence of the malarial parasite, which was identical with that in the blood of the patient upon whom the mosquitoes were fed in Rome.

We have, therefore, a most interesting pathological and therapeutic test of what was once a theory, and which is now a certainty. —*The Therapeutic Gazette.*

